# Salience of Medical Concepts of Inside Clinical Texts and Outside Medical Records for Referred Cardiovascular Patients

**DOI:** 10.1007/s41666-019-00044-5

**Published:** 2019-01-28

**Authors:** Sungrim Moon, Sijia Liu, David Chen, Yanshan Wang, Douglas L. Wood, Rajeev Chaudhry, Hongfang Liu, Paul Kingsbury

**Affiliations:** 1 Department of Health Sciences Research, Mayo Clinic, Rochester, MN USA; 2 Department of Computer Science and Engineering, University at Buffalo, Buffalo, NY USA; 3 Center For Innovation, Mayo Clinic, Rochester, MN USA; 4 Department of Medicine and Center for Translational Informatics, Mayo Clinic, Rochester, MN USA

**Keywords:** Outside medical records, Optical character recognition, Natural language processing, Electronic health record, Medical concept matching, Medical concept evaluation

## Abstract

Outside medical records (OMRs) accompanying referred patients are frequently sent as faxes from external healthcare providers. Accessing useful and relevant information from these OMRs in a timely manner is a challenging task due to a combination of the presence of machine-illegible information and the limited system interoperability inherent in healthcare. Little research has been done on investigating information in OMRs. This paper evaluated overlapping and non-overlapping medical concepts captured from digitally faxed OMRs for patients transferring to the Department of Cardiovascular Medicine and from clinical consultant notes generated at the Mayo Clinic. We used optical character recognition (OCR) techniques to make faxed OMRs machine-readable and used natural language processing (NLP) techniques to capture clinical concepts from both machine-readable OMRs and Mayo clinical notes. We measured the level of overlap in medical concepts between OMRs and Mayo clinical narratives in the quantitative approaches and assessed the salience of concepts specific to Cardiovascular Medicine by calculating the ratio of those mentioned concepts relative to an independent clinical corpus. Among the concepts collected from the OMRs, 11.19% of those were also present in the Mayo clinical narratives that were generated within the 3 months after their initial encounter at the Mayo Clinic. For those common concepts, 73.97% were identified in initial consultant notes (ICNs) and 26.03% were captured over subsequent follow-up consultant notes (FCNs). These findings implied that information collected from the OMRs is potentially informative for patient care, but some valuable information (additionally identified in FCNs) collected from the OMRs is not fully used in an earlier stage of the care process. The concepts collected from the ICNs have the highest salience to Cardiovascular Medicine (0.112) compared to concepts in OMRs and concepts in FCNs. Additionally, unique concepts captured in ICNs (unseen in OMRs or FCNs) carried the most salient information (0.094), which demonstrated that ICNs provided the most informative concepts for the care of transferred patients.

## Introduction

The “meaningful use” of electronic health records (EHRs) was called out by the HITECH (Health Information Technology for Economic and Clinical Health) Act as “the use of Health Information Technology that furthers the goals of information exchange among health care professionals” [1]. In theory, EHRs enable health information exchange (HIE) [2]. EHRs, however, are not fully interoperable in practice, and as such, the ability to digitally exchange information between providers is limited. As a result, digitally faxed scanned documents continue to be used to convey information between providers.

Retrieving clinically important information from digitally faxed outside medical records (OMRs) is, however, a non-trivial issue, as some patients who are seeking specialized care for chronic medical conditions from a tertiary care center can have a massive amount of OMRs. In the best case where the OMRs are properly handled, an expert (care coordinator or designated nurse) manually reviews the OMRs to identify the recent and relevant information prior to patient encounters. This review process demands significant resources in time and effort, and the chances for errors or lapses are significant. As a worst-case scenario, the incoming OMRs are superficially skimmed and useful information in OMRs can outright be missed or ignored at the point of care. This may contribute to inefficiency of care with respect to time and cost. Furthermore, the transmission of OMRs might be the only form of direct communication between primary care physicians and referral institutions [3], demonstrating the importance of OMRs in continuity of care. An open question is therefore how to accomplish the full utilization of information of OMRs from varying providers.

To make medical decisions promptly, accurate information must be readily accessible. The cost in human effort to process incoming scanned OMRs within a reasonable timeframe can be significantly mitigated using a combination of optical character recognition (OCR) and natural language processing (NLP). Specifically, OCR technology transforms a scanned image or a portable document format (PDF) file into searchable text. Using the output from the OCR process, NLP technologies can then extract the pertinent clinical information to form a summarization.

OCR in both handwritten and typewritten texts has been extensively studied in the domain of pattern recognition and computer vision. Prominent off-the-shelf OCR tools [4] include Google Cloud OCR,^1^ Tesseract,^2^ ABBYY FineReader,^3^ and Transym.^4^ All of these aforementioned software packages, with some configuration customization, can generate sufficiently machine-readable OCR output given a high-quality input image. OCR results, however, can be significantly dependent on scanning noise, page layout, and image resolution [5]. Techniques used by OCR engines in the medical domain are similar to those used in the general domain [6]. Results from these OCR technologies have been used to identify particular characters or digits from handwritten clinical documents [6–8], to obtain the value of data elements in a pre-designated area (e.g., checkbox or semi-structured table) of scanned forms [9–11], to reinforce supplementary information for EHRs [6, 8], or to retrieve relevant scanned documents [12, 13] from EHRs. A majority of these studies, however, collect a limited set of anticipated values (characters or digits) from homogenous data sources in particular EHR systems.

Independent from OCR research, information extraction from clinical narrative notes has been extensively explored in the clinical domain [14]. Information extraction starts from extracting events and concepts using well-known NLP systems such as MedLEE [15], MetaMap [16], cTAKES [17], and MedTagger [18]. These tools use various approaches to extract events and concepts, such as dictionary look-up using controlled vocabularies, rule-based methods applying pattern recognition, and machine learning such as Naïve Bayes or Support Vector Machine (SVM) techniques. In clinical practice, an aggregation of such relevant events and concepts can be used to deliver valuable information to clinicians as a summarization of EHRs. Diverse approaches [19–22] to extract clinical concepts and variables from clinical notes have been explored. The majority of these studies, however, did not evaluate extraction in a clinical practice setting, or their systems were not deployed [23] and relied on structured data sections of the EHR to discover major concepts. Only a limited number of studies [19] focused purely on unstructured texts.

There are a few existing studies using both OCR and NLP technologies for clinical applications. These studies applied additional NLP processes using limited medical terminologies or knowledge on top of OCR output to perform automated de-identification of patient information [24, 25], automated phenotyping or patient cohort identification from the EHR [6, 8, 26–28], and automated structured information generation through machine learning models [29].

For instance, a few systems constructed searchable texts from scanned paper-based EHR using OCR software (e.g., Tesseract). Personal health information (PHI) was then detected for pseudonymization purposes through pattern recognition and machine learning [24, 25]. The elements of interest were limited to PHI such as name, address, and identification numbers, rather than a broad range of semantics. Rasmussen and Peggy et al. [6, 8] detected supplemental evidence from scanned eye exam documents through the use of OCR (Tesseract) and NLP technologies (MedLEE) if their system could not find any conclusive evidence for cataract phenotype within the unstructured documents of the patient’s EHR. Similarly, Yadav et al. [27] classified traumatic brain injury (TBI) outcome by detecting TBI-related terminologies from scanned computed tomography image reports using MedLEE. Cui et al. [28] identified an epilepsy cohort through cTAKES by detecting terms denoting epilepsy and seizure in scanned discharge summaries. The coverage of these systems was, however, restricted to their respective medical domains, and these systems were mainly deployed on specialized types of reports, resulting in poor generalizability. Recently, a dictionary-based Chinese OCR pipeline was developed [29]. This system extracts medical concepts or entities (diagnosis, medication, and test) from images of structured reports (e.g., results of lab test or prescriptions). The majority of their research, however, relied on manual annotations, which requires a tremendous amount of effort, cost, and time. As a result, these studies have limited applicability to diverse practical settings at a large scale.

In this study, we seek to test the feasibility of a general-purpose OCR-to-NLP pipeline for OMRs. Specifically, we, first, automatically capture the level of overlapping medical concepts across OMRs from clinical consultant notes generated at the Mayo Clinic and, second, assess the utility of concepts for Cardiovascular Medicine. We first used the clinical consultant notes of patients referred to Cardiovascular Medicine for specialized care to calculate the coverage ratio of concepts extracted from scanned OMRs. We applied OCR and NLP technologies on available OMRs and clinical notes to extract relevant medical concepts/entities in three document sets: OMRs, the initial consultant notes (ICNs), and the follow-up consultant notes (FCNs) up to 3 months from ICNs. We then measured the information value (saliency scores) of overlapping and non-overlapping concepts for the requirements of Cardiovascular Medicine, using simple statistical measures compared to an independent cohort representing various specialty and practice settings at Mayo Clinic. Our study demonstrated the feasibility of automatically recognizing informative concepts from digitally faxed OMRs. Additionally, our proposed approach offers robust applicability in terms of the size of the cohort and generalizability across different practices due to the existence of an automated evaluation process.

## Materials

This study used two separate cohorts with appropriate approval from Mayo’s IRB (IRB 13-009317 and 18-001087). One cohort, the OMR cohort, is drawn from patients who visited the Mayo Clinic Department of Cardiovascular Medicine in spring 2016. In this study, we selected a total of 294 patients who have both OMRs from a diverse set of external providers, as scanned images in Adobe® portable document format (PDF) files, and clinical consultant notes generated at the Mayo Clinic Rochester campus.

We developed three document corpora for the patients in the OMR cohort. First, we formed an OMR corpus consisting of the OMRs for the patients in the cohort. Our OMRs were digital-faxed typewritten documents in PDF format. Nurse practitioners populated the metadata of these PDFs with document dates and document types (e.g., “note,” “echo reports,” “ECG reports”). We used Tesseract [30] to convert the PDFs to machine-readable data, in the form of raw text indexed by page number. On average, the patients in the OMR corpus have 40.55 pages of documents, totaling on average 8064.34 words per patient.

The second and third corpora for the patients in the OMR cohort were retrieved from their consultant notes in the Mayo Clinic EHR, where consultant notes are authored by consulting physicians to document critical clinical care information during patient encounters. The main usage of these notes is capturing (1) the past and current medical history of patients in detail and (2) the specific clinical decisions made alongside the thought process of the relevant physician. The second corpus was an “initial consultant note” (ICN) corpus containing consultant notes generated in the patient’s initial encounter at the Mayo Clinic. The third corpus, “follow-up consultant notes” (FCN), consisted of additional consultant notes generated within the 90 days (3 months) immediately subsequent to the patient’s initial encounter at the Mayo Clinic. The average number of clinical consultant notes per patient was 2.51 notes, totaling on average 1745.42 words per patient. In total, we collected 368 ICNs and 369 FCNs for this study.

Because the ICN and FCN corpora were expected to be skewed toward Cardiovascular Medicine, we collected a fourth corpus for use as a baseline comparison. The cohort for this independent corpus was the Employee and Community Health (ECH) cohort consisting of patients receiving primary care at Mayo Clinic in 2013 [31]. We collected all clinical notes created in 2016 for the ECH cohort across all clinical settings and specialties at Mayo Clinic. These collected clinical notes (ECN) included not only consultant notes but also any other type of clinical notes in the outpatient and inpatient settings at Mayo Clinic. About 1,470,000 ECNs were retrieved for about 95,000 patients in 771 different clinical practice settings. The purpose behind these four corpora was twofold. First, the OMR, ICN, and FCN corpora can be compared to discover how information flows into Mayo Clinic specialty care and whether that information is incorporated in subsequent care. The concepts found in the OMR, ICN, and FCN corpora can then be compared to the concepts found in the ECN corpus to discover the concepts that are most salient to the Cardiovascular Medicine setting at Mayo Clinic.

## Methods

**Fig. 1:**
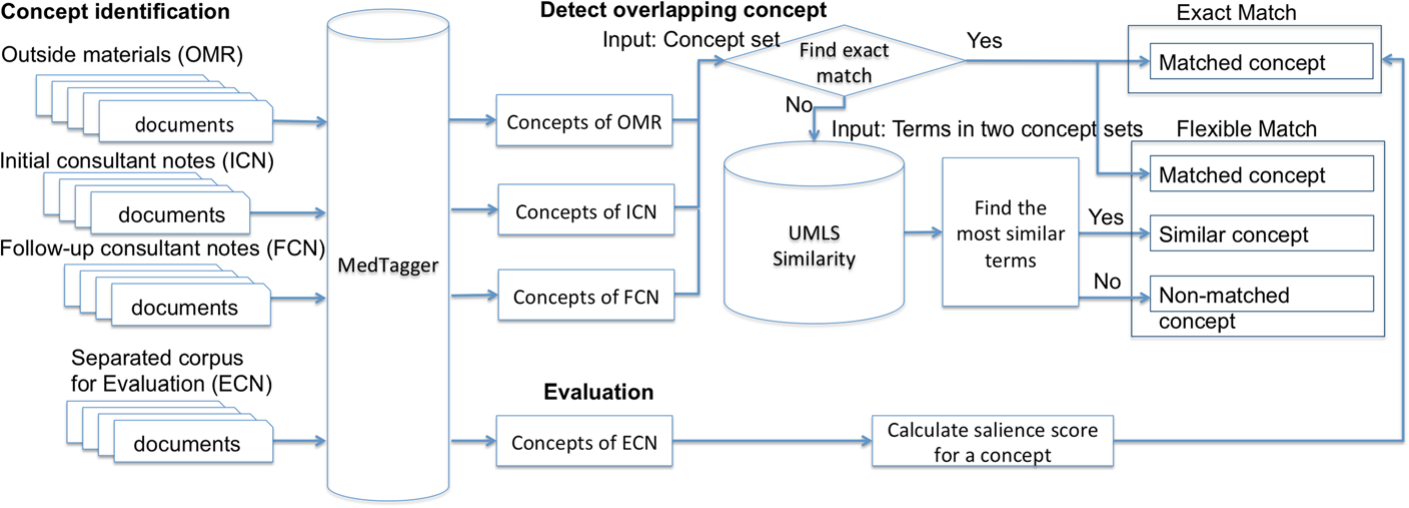
An overview of the processes of this study to identify, to map, and to evaluate concepts

**Fig. 2:**
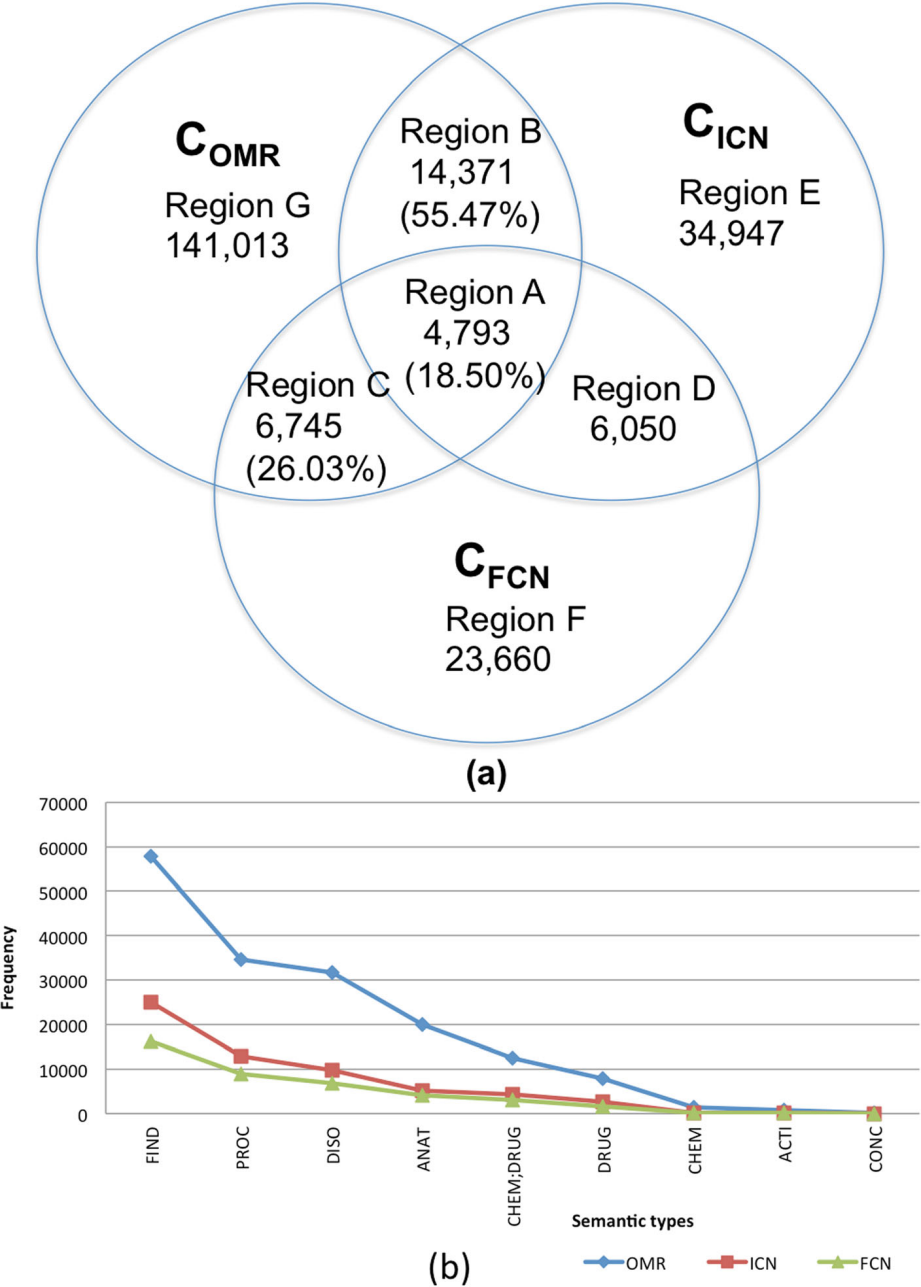
**a** The frequency and distribution of cardiovascular-specific concepts in *C*_OMR_, *C*_ICN_, and *C*_FCN_. **b** The distribution of semantic types for the identified concepts

In this section, we describe the development and usage of a medical concept extraction system on the aforementioned materials (OMRs and clinical texts) and an evaluation approach to calculate the salience of concepts to Cardiovascular Medicine. Figure 1 presents an overview of this study’s pipeline to identify, map, and evaluate concepts. Medical concepts and their corresponding semantic groups were extracted from text using an NLP tool for the four corpora: OMRs, ICNs, FCNs, and ECNs. We investigated the level of overlap between medical concepts in OMRs, ICNs, and FCNs. Any particular concept could appear in one, two, or all three corpora, so the three sets of concepts (one per corpus) could be divided into seven logical subsets (Fig. 2a). From here, we calculated the intersection ratio of overlapping concepts. Finally, we compared the concept frequencies in the three specialty corpora to the general ECN corpus, in order to assess the usage of concepts within Cardiovascular Medicine from the seven subsets.

### Corpus Identification

A key aspect of this study is the division between the ICNs and the FCNs, based upon the aforementioned 90-day window after the initial patient encounter at Mayo Clinic. While the Mayo notes themselves are clearly date-stamped, the relation between these notes and the specialty visit to Cardiovascular Medicine, and the remittance of the OMR corpus, was not always straightforward because the OMRs did not use consistent marking of dates. When possible, we utilized the dates identified by experts who manually populated the OMR metadata. If the date for a given patient was not available, we used the latest date from those identified among all the OMRs and the creation date of PDF files. This latest-created date among the OMRs could be compared to the dates on Mayo Clinic notes, and the first encounter date at Mayo after the latest date in the OMRs was used as the “initial encounter.” This served to eliminate visits related to other reasons or diseases.

As mentioned earlier, the ICN corpus was those notes created on the date of the “initial encounter” as defined in the previous paragraph. The ICN corpus may contain multiple consultant clinical notes per patient since one patient may visit different practice settings in the encounter day. The FCN corpus was all notes created between 1 and 90 days after that “initial encounter.”

### Concept Identification

We extracted medical concepts in texts (OMRs and clinical notes) using MedTagger [32]. Due to the complexity of natural language, one medical concept may have diverse mentions in clinical documents. For example, physicians can use distinct expressions such as “structure of heart” or “cardiac” to indicate the concept “heart.” To overcome this challenge, we used the Unified Medical Language System (UMLS),^5^ a terminology resource in the biomedical and clinical domain. In the above instance, “structure of heart” and “cardiac” were assigned a Concept Unique Identifier (CUI) of C0018787 with the preferred term “heart” in the UMLS. Using MedTagger, a clinical information extraction tool based on NLP, various expressions associated with the same UMLS concept were normalized to the same CUI. MedTagger generates additional contextual attributions (such as assertion, temporality, and experiencer) for each extracted concept. For example, “Patient had a history of heart attack” shows an experiencer attribute of “Patient,” whereas “His father had a history of heart attack” has an experiencer of “Others.” For our study, since we sought to understand the status only of the referred patient, we focused on concepts associated with “Patient” as experiencer.

Additionally, MedTagger divides those normalized terms into pertinent semantic types according to the UMLS. Several semantic types in the UMLS are pertinent to this study, including Activities & Behaviors (ACTI), Anatomy (ANAT), Chemicals (CHEM), Concepts & Ideas (CONC), Disorders (DISO), Drug (DRUG), Finding (FIND) and Procedures (PROC). On the one hand, since the concept may belong to multiple semantic types, this semantic group can be represented as a semicolon-delimited value set. As an example, “warfarin” corresponds to multiple semantic types including CHEM and DRUG, resulting in a recorded semantic type of “CHEM;DRUG.” On the other hand, a normalized term in clinical notes can have differing semantic types depending on the context. As an example, “heart” corresponds to DISO as the semantic type in “Hospitalization in March 2015 for heart failure” but ANAT as the semantic type in “The patient’s tachycardia is in response to her PE with mild right cardiac strain.”

We then aggregated all extracted concepts with corresponding details into the defined sets: OMRs, ICNs, FCNs, and ECNs. Corresponding details were associated semantic type and unique clinical number of patient. For instance, running MedTagger on the sentence “There is also evidence of moderate coronary vascular calcifications on the CT” generates two independent concepts for “CT.” One is “chest xray::FIND::0000” and the other is “chest xray::PROC::0000” (where “0000” is a dummy patient clinical number for example purposes). Concepts consist of normalized terms with corresponding details in our study. We collected a set of concepts${C}_{\mathrm{OMR}}=\left\{{c}_{\mathrm{OMR}}^1,{c}_{\mathrm{OMR}}^2,\dots, {c}_{\mathrm{OMR}}^N\right\}$ from OMRs. Similarly, from clinical notes, we built${C}_{\mathrm{ICN}}=\left\{{c}_{\mathrm{ICN}}^1,{c}_{\mathrm{ICN}}^2,\dots, {c}_{\mathrm{ICN}}^N\right\}$, ${C}_{\mathrm{FCN}}=\left\{{c}_{\mathrm{FCN}}^1,{c}_{\mathrm{FCN}}^2,\dots, {c}_{\mathrm{FCN}}^N\right\}$, and ${C}_{\mathrm{ECN}}=\left\{{c}_{\mathrm{ECN}}^1,{c}_{\mathrm{ECN}}^2,\dots, {c}_{\mathrm{ECN}}^N\right\}$, where *c* denotes a concept from texts. We regarded the combined extracted concept information from ICNs and FCNs as the “gold standard” for patient care.

### Concept Matching

We applied two levels of matching while matching concepts from OMRs to EHRs: exact matching and flexible matching. While exact matching only considers concepts with the same normalized form as a valid match, flexible matching also considers the concepts with high semantic similarity. The similarity scores are calculated between extracted concepts in separate sets. We considered the set pairs (*C*_OMR_ and *C*_ICN_), (*C*_OMR_ and *C*_FCN_), and (*C*_OMR_ and *C*_CN_) where *C*_CN_ denotes the union of (*C*_ICN_ and *C*_FCN_). For exact-matching concepts, the intersection ratio *r*_*e*_ is defined as follows:

1 $$ {r}_e=\frac{\mid {C}_{\mathrm{OMR}}\cap {C}_{\mathrm{CN}}\mid }{\mid {C}_{\mathrm{CN}}\mid } $$

As semantic granularity is imbalanced in the UMLS [33, 34], we also considered flexible matching to map closely relevant concepts. For this task, we use the UMLS similarity score of two UMLS concepts as provided by the “UMLS-Similarity” [35] package. This UMLS similarity is calculated by identifying UMLS concepts associated to the normalized terms provided as input, measuring the semantic similarity of those identified concepts using one of a number of metrics, and returning the similarity score as output for each pair of concepts. We chose the path measure [36], which is the default similarity measure of the package. The similarity score, sim(*c*_*i*_, *c*_*j*_), is calculated as follows:

2 $$ \mathrm{sim}\left({c}_i,{c}_j\right)=\frac{1}{l\left({c}_i,{c}_j\right)} $$

where *l*(*c*_*i*_, *c*_*j*_) indicates the minimum number of nodes between *c*_*i*_ and *c*_*j*_ (the shortest path) in the UMLS. The shortest possible path contains only one node, when the two concepts are identical. If any one of the two items in the normalized term pairs cannot be mapped to a UMLS concept or the two concepts have no relationship in the UMLS, it yields − 1 for the similarity score. Otherwise, similarity scores between *c*_*i*_ and *c*_*j*_ range from 0 to 1, with scores near 0 denoting little to no similarity, and 1 denoting that the two concepts are the same. Mathematically, the range of sim(*c*_*i*_, *c*_*j*_) can be defined as (0, 1] ∩ {−1}.

We calculated pair-wise similarity scores between all concepts in *C*_CN_ and *C*_OMR_. For each concept in *C*_OMR_, we chose the concept in *C*_CN_ with the highest similarity score to form *C*_SIMILAR_ if applicable.

The similar intersection ratio *r*_*s*_ is defined as:

3 $$ {r}_s=\frac{\mid {C}_{\mathrm{SIMILAR}}\mid }{\mid {C}_{\mathrm{CN}}\mid } $$

### Evaluation

We consider high intersection ratios to reflect high information utilization from OMRs. We assess the information overlap for each of the seven logical regions on concurrently appearing concepts in overlapping and non-overlapping corpora:

- 4 $$ \mathrm{Region}\ \mathrm{A}=\left(\ {C}_{\mathrm{OMR}}\cap {C}_{\mathrm{ICN}}\cap {C}_{\mathrm{FCN}}\right):\mathrm{the}\ \mathrm{concept}\ \mathrm{set}\ \mathrm{present}\ \mathrm{in}\ \mathrm{all}\ \mathrm{three}\ \mathrm{corpora}\ \left(\mathrm{OMRs},\mathrm{ICNs},\mathrm{and}\ \mathrm{FCNs}\right) $$
- 5 $$ \mathrm{Region}\ \mathrm{B}=\left({C}_{\mathrm{OMR}}\cap {C}_{\mathrm{ICN}}\right)-\left(\ {C}_{\mathrm{OMR}}\cap {C}_{\mathrm{ICN}}\cap {C}_{\mathrm{FCN}}\right):\mathrm{the}\ \mathrm{concept}\ \mathrm{set}\ \mathrm{present}\ \mathrm{in}\ \mathrm{OMRs}\ \mathrm{and}\ \mathrm{ICNs}\ \mathrm{but}\ \mathrm{absent}\ \mathrm{in}\ \mathrm{FCNs} $$
- 6 $$ \mathrm{Region}\ \mathrm{C}=\left({C}_{\mathrm{OMR}}\cap {C}_{\mathrm{FCN}}\right)-\left(\ {C}_{\mathrm{OMR}}\cap {C}_{\mathrm{ICN}}\cap {C}_{\mathrm{FCN}}\right):\mathrm{the}\ \mathrm{concept}\ \mathrm{set}\ \mathrm{present}\ \mathrm{in}\ \mathrm{OMRs}\ \mathrm{and}\ \mathrm{FCNs}\ \mathrm{but}\ \mathrm{absent}\ \mathrm{in}\ \mathrm{ICNs} $$
- 7 $$ \mathrm{Region}\ \mathrm{D}=\left({C}_{\mathrm{ICN}}\cap {C}_{\mathrm{FCN}}\right)-\left(\ {C}_{\mathrm{OMR}}\cap {C}_{\mathrm{ICN}}\cap {C}_{\mathrm{FCN}}\right):\mathrm{the}\ \mathrm{concept}\ \mathrm{set}\ \mathrm{present}\ \mathrm{in}\ \mathrm{ICNs}\ \mathrm{and}\ \mathrm{FCNs}\ \mathrm{but}\ \mathrm{absent}\ \mathrm{in}\ \mathrm{OMRs} $$
- 8 $$ \mathrm{Region}\ \mathrm{E}={C}_{\mathrm{ICN}}-\left(\ {C}_{\mathrm{OMR}}\cup {C}_{\mathrm{FCN}}\right):\mathrm{the}\ \mathrm{set}\ \mathrm{of}\ \mathrm{concepts}\ \mathrm{present}\ \mathrm{in}\ \mathrm{ICNs}\ \mathrm{but}\ \mathrm{absent}\ \mathrm{in}\ \mathrm{OMRs}\ \mathrm{or}\ \mathrm{FCNs} $$
- 9 $$ \mathrm{Region}\ \mathrm{F}={C}_{\mathrm{FCN}}-\left(\ {C}_{\mathrm{OMR}}\cup {C}_{\mathrm{ICN}}\right):\mathrm{the}\ \mathrm{set}\ \mathrm{of}\ \mathrm{concepts}\ \mathrm{present}\ \mathrm{in}\ \mathrm{FCNs}\ \mathrm{but}\ \mathrm{absent}\ \mathrm{in}\ \mathrm{OMRs}\ \mathrm{or}\ \mathrm{ICNs} $$
- 10 $$ \mathrm{Region}\ \mathrm{G}={C}_{\mathrm{OMR}}-\left(\ {C}_{\mathrm{ICN}}\cup {C}_{\mathrm{FCN}}\right):\mathrm{the}\ \mathrm{set}\ \mathrm{of}\ \mathrm{concepts}\ \mathrm{present}\ \mathrm{in}\ \mathrm{OMRs}\ \mathrm{but}\ \mathrm{absent}\ \mathrm{in}\ \mathrm{ICNs}\ \mathrm{or}\ \mathrm{FCNs} $$

The overlapping concepts between OMRs and ICNs were represented in the combination of region A and region B. The overlapping concepts between OMRs and FCNs (but absent in ICNs) were the combination of region A and region C. We then interpreted region A, region B, and region C as follows: region A represented identified information from OMRs that was thoroughly incorporated and used throughout the course of the patient’s care at the Mayo Clinic. Region B is the set of the presented information from OMRs that were utilized during the initial encounter, but discarded in follow-up visits at Mayo Clinic. Region C contains information presented in the patient’s OMRs, but were ignored or overlooked during the initial consultation, to be eventually rediscovered and incorporated in follow-up encounters.

Region D, region E, and region F represent concepts newly discovered at Mayo Clinic. Specifically, region D contains new concepts from both ICNs and FCNs. The concepts of region E represent identified information at Mayo Clinic (ICNs) but discarded in FCNs, whereas the concepts in region F indicate new information appearing during follow-up that was unknown in both OMRs and ICNs. Additionally, region G represents information that was not incorporated at Mayo Clinic but was available in OMRs.

To identify key concepts in Cardiovascular Medicine, we calculated the mentioned level of collected concepts over all clinical practice settings at Mayo Clinic. If a particular concept (e.g., “cardiac arrest”) appears more frequently or exclusively in Cardiovascular Medicine compared to the overall practice, this concept may carry more specialty-specific information. On the other hand, an infrequent or absent concept (e.g., “generalized anxiety disorder”) may convey clinical information not important for Cardiovascular Medicine. Therefore, the ratio of the frequencies implies the salience of concepts specific to a practice. For each concept in the ECNs identified by MedTagger, we counted its frequency first in clinical notes specific to Cardiovascular Medicine, and then its frequency in all departments and specialties represented in the ECNs. Our saliency score of a concept from the perspective of Cardiovascular Medicine is defined as:

- 11 $$ \mathrm{Saliency}\ \mathrm{score}\ \mathrm{for}\ \mathrm{a}\ \mathrm{concept}=\frac{\mathrm{Frequency}\ \mathrm{of}\ {C}_{\mathrm{CN}}\ \mathrm{in}\ \mathrm{Cardiovascular}\ \mathrm{Medicine}}{\mathrm{Frequency}\ \mathrm{of}\ {C}_{\mathrm{CN}}\ \mathrm{in}\ \mathrm{overall}\ \mathrm{practical}\ \mathrm{settings}} $$
- 12 $$ \mathrm{Average}\ \mathrm{saliency}\ \mathrm{score}\ \mathrm{for}\ \mathrm{one}\ \mathrm{region}=\frac{\sum \mathrm{saliency}\ \mathrm{score}\ \mathrm{of}\ \mathrm{concept}\ \mathrm{in}\ \mathrm{the}\ \mathrm{region}}{\mid \mathrm{saliency}\ \mathrm{score}\ \mathrm{of}\ \mathrm{concept}>0\ \mathrm{in}\ \mathrm{the}\ \mathrm{region}\mid } $$

Collected saliency scores of all identified concepts in Cardiovascular Medicine were ranked in descending order to distinguish key concepts. The maximum saliency score is 1, which represents a concept exclusively appearing in clinical notes generated by Cardiovascular Medicine. If the score is close to 0, the concept is commonly discussed in all practice settings, or used rarely in Cardiovascular Medicine. To represent the salience of concepts in the regions defined earlier (Eq. 4–Eq. 10) in our study, we calculated the average saliency score by summing up all scores for available concepts, and then dividing by the number of available concepts with positive scores.

## Results

Across the three corpora, we identified 231,579 unique concepts in total. Of these, OMRs contained 166,922 unique concepts, ICNs contained 60,161 unique concepts, and FCNs had 41,248 unique concepts. With respect to the set of unique concepts, the preponderance (72.08%) belonged to *C*_OMR_ and 11.19% (25,909 concepts and 15.52% of the total *C*_OMR_) overlap between OMRs and clinical notes in Mayo Clinic. Of these overlapping concepts, *C*_ICN_ contained 73.97% while *C*_FCN_ contained 44.53%. Note that the incidence rate of overlapping concepts in *C*_ICN_ is higher than in *C*_FCN_ despite *C*_ICN_ and *C*_FCN_ having a similar number of clinical notes.

To calculate the salience of concepts specific to Cardiovascular Medicine, we used all concepts from ECNs in all practice settings. For the evaluation set from ECH cohorts, 2.13% of ECNs (about 31,000 CNs for 4876 patients) belonged to Cardiovascular Medicine. MedTagger generated about 113,554,000 concepts in total from ECNs, of which 2.94% (3,343,000 concepts) were relevant to Cardiovascular Medicine. Additionally, CNs (including ICNs and FCNs) contain a total 31,261 unique concepts in Cardiovascular Medicine (29 practice settings [37]).

Figure 2a visualizes the frequency of unique concepts in each of the regions in the form of a Venn diagram, where Region A (*n* = 4793), Region B (*n* = 14,371) and Region C (*n* = 6745) contributed 25,909 overlapping concepts. The distribution of the total collected semantic types associated with identified concepts used by MedTagger is presented in Fig. 2b. The overlapping concept sets (region A, region B, and region C) have analogous patterns of semantic types in terms of distribution. The dominant semantic types are FIND (40.45%), PROC (21.75%), DISO (16.35%), ANAT (9.98%), CHEM;DRUG (7.51%), and DRUG (3.55%) over the set region A ∪region B∪ region C. These overlapping concepts covered 28.61% of the total identified concepts in *C*_ICN_∪ *C*_*FCN*_.

The concept coverage of CNs (ICNs or FCNs) from OMRs with regard to semantic types is shown in Table 1. In Table 1, exact match is Eq. 1 and flexible match is Eq. 3. Overall, *C*_ICN_ has more overlapping concepts with *C*_OMR_ compared to *C*_OMR_ ∩ *C*_FCN_. This pattern was found across the other major semantic types. In detail, *C*_ICN_ when Eq. 1 (the exact matching method) was used, 31.85% of the total concepts in *C*_OMR_ ∩ *C*_ICN_ were overlapping, while *C*_FCN_ contained 27.97% of overlapping concepts in *C*_OMR_ ∩ *C*_FCN_. This implies that OMRs share more concepts with ICNs than with FCNs. Among identified semantic types, CHEM;DRUG had the highest overlapping ratio in *C*_OMR_ ∩ *C*_ICN_, and in *C*_*OMR*_ ∩ *C*_*FCN*_ when using exact match. This indicates that CNs (ICNs and FCNs) and OMRs used identical terminologies to capture current medication or lab test information. DISO was ranked as third (after ANAT) in frequency of overlapping semantic types between *C*_ICN_ and *C*_OMR_ while FIND (after ANAT) is the third highest frequency semantic type overlap between *C*_FCN_ and *C*_OMR_. This implies that ICNs contained the majority of disease information while FCNs had additional information (such as a clinical observation or measurement) for a given disease. When using Eq. 3 (the flexible matching method), both sets increased the overlap ratio about 15~17% compared to the exact matching method using Eq. 1. The set benefitting the most in terms of overlap ratio improvement is DISO when using Eq. 3 (the flexible matching method). This indicates CNs use diverse expressions for diseases and suggests that detection of relevant terms or clinical phrases could improve the utilization of OMR information.

**Table 1:** Concept coverage ratio to outside medical records

Major semantic type	Concept coverage ratio to ***C***_**OMR**_			
	***C***_***ICN***_		***C***_***FCN***_	
	Exact match (%)	Flexible match (%)	Exact match (%)	Flexible match (%)
CHEM;DRUG	34.94	51.08	31.06	43.14
ANAT	34.92	53.74	29.37	47.32
DISO	32.60	57.05	27.86	48.31
PROC	31.88	50.56	27.29	43.98
CHEM	30.94	48.92	25.23	40.54
FIND	30.93	44.50	27.97	39.73
DRUG	27.47	37.68	24.30	32.52
ACTI	25.00	28.75	15.58	22.08
CONC	18.92	18.92	25.00	25.00
Total	31.85	48.78	27.97	42.76

**Fig. 3:**
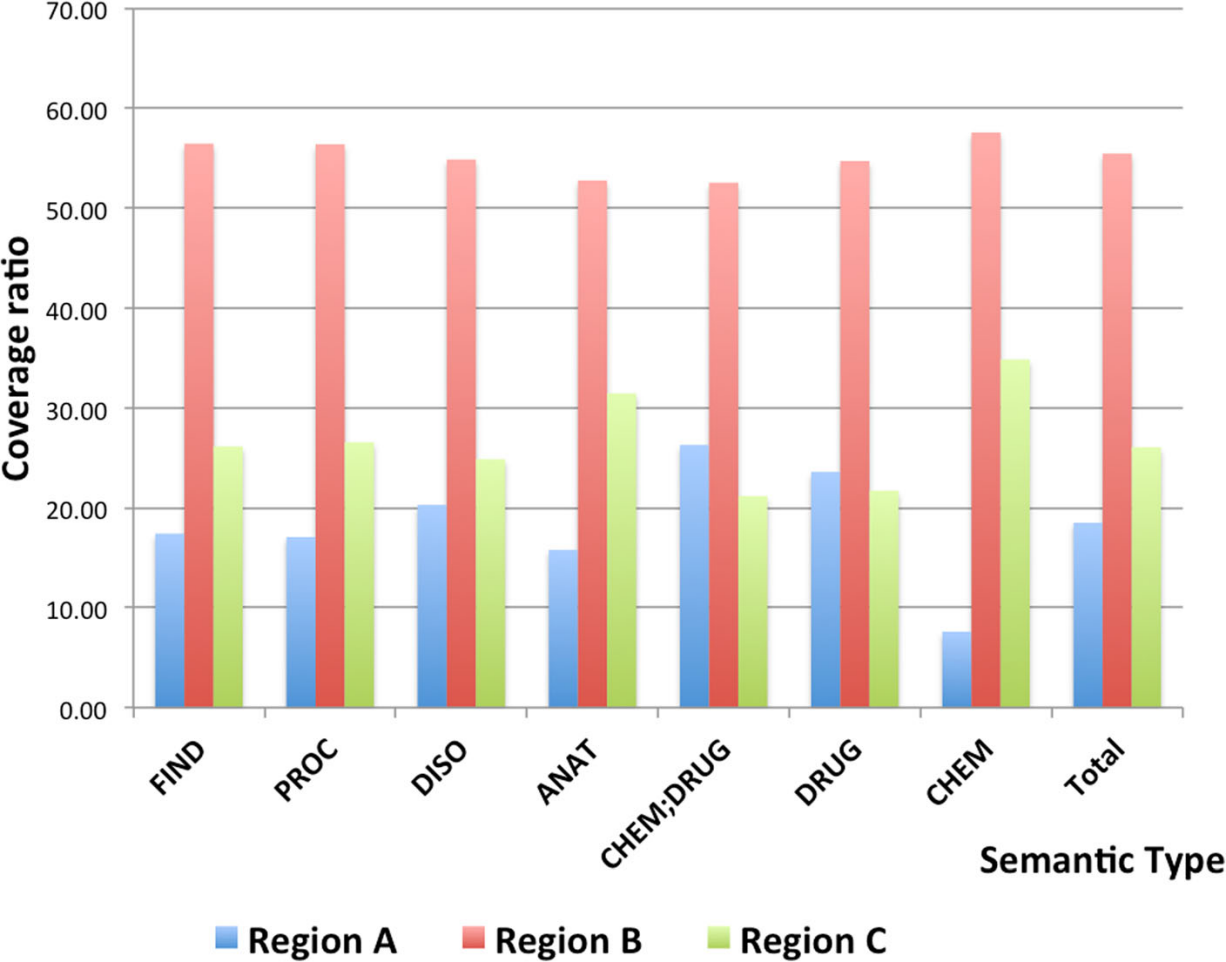
Concept coverage ratios over the total overlapping concepts between OMRs and CNs, with respect to semantic types

Common concepts in OMRs and Mayo clinical notes (the union of region A, region B, and region C) when using the exact matching method (Eq. 1) are shown in Table 2 and Fig. 3. The concept coverage ratio is defined as the frequency of detected concepts of each semantic type for each region divided by the total frequency of those in Mayo clinical notes (the union of region A, region B, and region C). Focusing on the semantic types of concepts shared by OMRs and Mayo clinical notes, we see that FIND is the predominant semantic type in each region, followed in order by PROC, DISO, and ANAT.

**Table 2:** Concept coverage ratio to the intersection of concepts of outside medical records

Major semantic type	Concept coverage ratio to region A**∪** region B **∪** region C						
	Region A		Region B		Region C		Total
	Frequency	Ratio%	Frequency	Ratio%	Frequency	Ratio%	Frequency
FIND	1825	17.42	5917	56.47	2737	26.12	10,479
PROC	962	17.07	3178	56.40	1495	26.53	5635
DISO	860	20.30	2325	54.87	1052	24.83	4237
ANAT	408	15.78	1364	52.77	813	31.45	2585
CHEM;DRUG	511	26.27	1022	52.54	412	21.18	1945
DRUG	217	23.56	504	54.72	200	21.72	921
Total	4793	18.50	14,371	55.47	6745	26.03	25,909

The largest portion of overlapping concepts, about 55.47% in Fig. 2a, belongs to region B (the set present in OMRs and ICNs but absent in FCNs). This phenomenon is repeated for all the major semantic types, as seen in Fig. 3. Region C contained 26.03% of the total overlapping concepts while region A had 18.50%. The significance of an individual semantic type within a certain region was calculated as the percentage of that semantic type over all semantic types present in that particular region. For example, in Table 2, ANAT was underrepresented in region A since only 15.78% of ANAT appearing as compared to the average semantic types was 18.50% in region A. CHEM;DRUG was overrepresented in region A with 26.27% of those concepts. On the other hand, ANAT was overrepresented, whereas CHEM;DRUG was underrepresented in region C.

The distribution of common concepts of OMRs and Mayo clinical notes can be further investigated with respect to 12 major different sections in clinical notes at Mayo Clinic, specifically “Allergy,” “Chief Complaint and reason for visit,” “Current Medications,” “Diagnosis,” “Family History,” “History of present illness,” “Impression/report/plan,” “Past medical and surgical history,” “Physical Exam,” “Social History,” “System Review,” and “Vital Signs.” We included multiple note sections per concept since one concept could appear in several sections in a single CN concurrently. For instance, “Tachy brady syndrome:DISO:0000” can appear in both “Diagnosis” and “Impression/report/plan” sections. We identified a total of 107,449 note sections with corresponding concepts in *C*_ICN_ ∪ *C*_FCN_. Of these detected corresponding concepts, the frequency order of sections with identified concepts was “Impression/report/plan” (containing 31.88% of detected concepts), “History of present illness” (27.19%), and “Physical Exam” (11.89%). “Impression/report/plan” preserved the major coverage of semantic types such as 40.13% of total PROCs, 31.35% of total ANATs, 29.80% of total FINDs, and 29.59% of total DISOs. “Current Medications” covered 31.08% of total concepts of DRUGs and 27.31% of CHEM;DRUG.

We examined in further detail the common concepts over OMRs (region A, region B, and region C in Fig. 2a) over concepts in *C*_ICN_ ∪ *C*_FCN_ (region A, region B, region C, region D, region E, and region F in Fig. 2a). We observed both union areas had the dominant note sections as “Impression/report/plan,” “History of present illness,” and “Physical Exam.” In contrast, certain information shifts from the section of “Current Medication” (4.82% of total note sections in *C*_ICN_ ∪ *C*_FCN_) to “Diagnosis” (4.83% of total note sections in region A ∪ region B ∪ region C). In terms of overlapping concepts, information shifts from “Diagnosis” in *C*_ICN_ (5.42% of total identified concepts in *C*_ICN_) to “Physical Exam” in *C*_FCN_(11.57% of total identified concepts in *C*_FCN_). These tendencies were confirmed when we reviewed the frequencies of sections in region A. The total frequency of concepts for region A is 4793 (Fig. 2a), and the frequencies of sections for *C*_ICN_(total 6963 sections for 4793 concepts) and those for *C*_FCN_(total 6395 sections for the same concept sets) were different from each other. *C*_ICN_ contained an additional 236 cases of “Diagnosis” and an additional 38 cases of “Physical Exam” compared to *C*_FCN_. In other words, some concepts appeared in the “Diagnosis” section in ICNs only, but later re-appeared in the “Physical Exam” section in FCNs since the diagnosis was verified through further examination. When we took region A, region B, and region C independently, “History of present illness” is a major section for region A, but “Impression/report/plan” is the predominant section for region B or region C. In other words, the concepts within the “History of present illness” section displayed less variability in both ICNs and FCNs due to repeated information of history illness. However, differing concepts may appear in the “Impression/report/plan” section between ICNs to FCNs due to additional information being gathered during examination after the initial consultation.

We investigated further the salience of concepts (Eq. 11–Eq. 12) for Cardiovascular Medicine for our collected concepts relative to independent ECNs in Table 3. Table 3 presents mapping ratios to ECNs and the average saliency scores in our regions of interest (Eq. 4–Eq. 10). OMRs, ICNs, and FCNs had average saliency scores of concepts as 0.066, 0.112, and 0.053, respectively. According to Table 3, the highest average saliency score is seen in region E, which represents concepts appearing in ICNs but not in OMRs and FCNs. All other regions (region A, region B, and region D) in ICNs had following high average saliency scores overall. These findings indicated that ICNs contained more necessary information (concepts in our study) for patient care with respect to Cardiovascular Medicine than OMRs or FCNs. Meanwhile, original OMRs had the highest frequency for unique concepts (*n* = 157,205). However, they had lower average saliency scores than those of ICNs since region G had the lowest mapping ratio (93.15%) to ECNs (*n* = 131,352) from original region G (*n* = 141,013). In other words, certain concepts in OMRs are less relevant to the care of Cardiovascular Medicine at Mayo Clinic. Similarly, region F also presented low coverage (96.95%), which contributed the lowest average saliency score in FCNs. Lastly, region A had the third highest average saliency score, even though the mapping frequency to ECNs was significantly low (*n* = 4779). Therefore, region A indicated highly dense, essential information for care in Cardiovascular Medicine.

**Table 3:** The mapping concepts and salience score to cardiovascular medicine

	Mapping concepts to ECNs	Coverage ratio to original set	Sum of salience score	Average salience score	Top 5 salient concepts (score)
Region A	4779	99.71%	411.25	0.086	antiarrhythmic::DRUG (0.749) coronary angiography::PROC (0.677) single ventricle::DISO (0.676) dofetilide::CHEM;DRUG (0.658) biventricular pacing::PROC (0.658)
Region B	14,338	99.77%	1266.28	0.088	d transposition of the great vessels::DISO (1.000) hv interval::FIND (1.000) isuprel::CHEM;DRUG (0.906) ventriculogram::PROC (0.810) multaq::CHEM;DRUG (0.800)
Region C	6736	99.87%	431.34	0.064	ventricular tachycardia symptomatic::DISO (1.000) qrs duration::FIND (0.777) leads v1::ANAT (0.770) respiratory exchange ratio::FIND (0.754) antiarrhythmic::DRUG (0.749)
Region D	5999	99.16%	484.62	0.081	absent pulmonary valve syndrome::DISO (1.000) waterston shunt::PROC (0.875) pulmonary vein stenosis::DISO (0.855) left ventricular noncompaction::DISO (0.800) catheter ablation for atrial fibrillation::PROC (0.783)
Region E	22,939	98.66%	3232.15	0.094	downward slanting palpebral fissures::FIND (1.000) injury pulmonary vein::DISO (1.000) catheter ablation for atrial arrhythmia::PROC (1.000) left superior vena cava::ANAT (1.000) Additional 6 DISO, 10 FIND, 2 PROC and 1 ANAT (1.000)
Region F	34,480	96.95%	1443.17	0.063	pulmonary vascular resistance index::FIND (1.000) fistula pulmonary::DISO (1.000) hv interval::FIND (1.000) sinoatrial node::ANAT (1.000) chronic constrictive pericarditis::DISO (1.000)
Region G	131,352	93.15%	8219.96	0.063	qs pattern::FIND (1.000) aortic root angiography::PROC (1.000) posterior fascicular block::DISO (1.000) inferior pulmonary vein::ANAT (1.000) Additional 22 FIND, 10 PROC, 8 DISO, 5 ANAT, 3 CHEM;DRUG, 1 DRUG (1.000)

## Discussion

In this study, concepts from OMRs and Mayo clinical notes were automatically identified, and extracted concepts were explored with respect to corpus overlap and salience. First, we investigated the overlap of information among OMRs and clinical notes (ICNs and FCNs). Second, we evaluated the salience of concepts for Cardiovascular Medicine using the frequency of concepts compared to concept statistics collected from a large independent clinical corpus at Mayo Clinic. We found that 11.19% of concepts in OMRs could also be found in clinical notes generated at the Mayo Clinic. Within this common information, we investigated two types of information as follows: (1) the continuity of available information and (2) omitted or additional information within 90 days from the initial encounter at Mayo Clinic. We identified 73.97% of the common information was seamlessly conveyed at the initial encounter. This finding indicates information from the OMRs was highly utilized. Nevertheless, this also suggests that 26.03% of overlapping concepts (from FCNs) were unavailable information from ICNs, and certain valuable information present in OMRs was omitted. To interpret the usage of concepts in Cardiovascular Medicine, additional analysis was conducted to calculate frequency of concepts used by Cardiovascular Medicine relative to the frequency of the same concepts over the entire clinical practice. Through this analysis, we found that the additionally identified information in ICNs is highly informative (average saliency score = 0.094) for patient care. In contrast, omitted OMRs information (average saliency score = 0.063) represented information not relevant for care in Cardiovascular Medicine at Mayo Clinic. Our study demonstrated the feasibility of capturing informative concepts from digitally faxed OMRs automatically without undue delay. Our automatic approach can be easily adopted at a larger scale or in the different practice settings in the future.

Using the overlapping concept sets between OMRs and CNs and by reviewing subsequent CNs of the patients beyond the ICNs, we further investigated the reasons for information to be present in FCNs but omitted in ICNs (region C in Fig. 2a). The major reasons were as follows: (1) the limitation of concept scope relevant to cardiovascular diseases of patients at the initial consultation: patients regularly suffer from comorbidities, requiring visits to different departments (e.g., endocrinology, psychology, etc.). Patients, in their initial consultation with Cardiovascular Medicine, tend to not discuss their conditions not related to CV, such as “generalized anxiety disorder” or “chronic periodontitis.” (2) Follow-up consultations will expand upon findings and complaints discovered during the initial consultations. ICNs hold condensed summarizations of “Social history” such as “She has never smoked. She is married.” On the other hand, FCNs include supplemental details such as “level of education,” “issues affecting learning,”, “employment status,” “alcohol,” “caffeine,” and others. (3) Both consultants and patients may not discuss peripherally related medical information in the initial encounter. As an example, a pre-existing condition (cirrhosis) of one patient was not addressed in the initial consultation but it was mentioned in the subsequent consultant notes.

We further investigated why information contained in OMRs was omitted from CNs at Mayo Clinic (region G in Fig. 2a). We hypothesize three potential reasons. First, a large number of patients receive a wide range of diagnostic tests to determine the cause of problems. Not all tests performed are guaranteed to be relevant to the final diagnosis. Irrelevant tests may contribute to additional noise and unnecessary information within OMRs. Since Mayo Clinic is a tertiary referral institution accustomed to complex cases, its specialists have access to more specialized diagnostic testing and have more experience with uncommon diseases and presentation of diseases, resulting in a faster and more definitive diagnosis. Therefore, unnecessary results (tests and examinations) and outdated information may be screened out at the beginning of care. This hypothesis is supported by the high density of information in referral CNs in Mayo Clinic compared to OMRs. Our results are consistent with a previous study by Sohn et al. [38]. In their study, semantic and concept information between the Mayo Clinic and Sanford Children’s Hospital (SCH) corpora for Pediatric Asthma were relatively homogenous; however, the density of salient concepts (i.e., those critical for decision-making) in the Mayo Clinic clinical notes is higher than that in the SCH notes.

The second reason is potential differences between the referral diagnosis in OMRs and the final diagnosis in CNs at Mayo Clinic. According to another case study on primary care practices [39], 88% of patients transferred from outside hospitals have a new or refined diagnosis at Mayo Clinic. Accurate diagnosis of many diseases is difficult due to similarity in signs and symptoms. One patient in this study came to Mayo Clinic to seek a second opinion related to a diagnosis of “arrhythmogenic right ventricular dysplasia (ARVD)” from an outside hospital. However, this patient had no evidence of ARVD after several examinations (a case of referral misdiagnosis). As another example, a patient was diagnosed with “acute exacerbation of chronic obstructive pulmonary disease (COPD)” by an outside hospital. However, upon further examination, this diagnosis was defined as “acute respiratory distress syndrome (ARDS).”

Lastly, there are time gaps between the receipt of OMRs and patient visits for consultation at Mayo Clinic. In our study, 40.14% of patients had clinical notes in Mayo Clinic prior to our ICNs while others had further outside examinations even after the initial consultation. Since not all OMRs arrive in time for the initial patient consultation at Mayo, patients receive new tests or examinations without OMRs, and then consultants discard the information of the tardy OMRs that are no longer clinically relevant or trustworthy for diagnosis at the time of the consult. And a majority of these patients re-visit Mayo Clinic over a relatively long period of time (1 year in this case), while taking their standard continuous care at outside primary providers.

This study has a scope limited to the use of OMRs at Mayo Clinic. We evaluated the usage of OMRs with reference to the limited number of patients within the Department of Cardiovascular Medicine. Additionally, our study performed the analysis regarding to concept identification with the lack of validation of OCR technologies. Our evaluation relied on concept frequencies with a lack of consideration for infrequent terminologies (i.e., rare disease [40]), and the lack of a ground truth dataset prevents direct comparisons with previous research. Nevertheless, we highlighted the fact that some of the available information in OMRs is not integrated in a timely manner into subsequent medical record of a tertiary care center, leading to the implication that patients are undergoing unnecessary or redundant procedures to produce clinical values that have already been documented in the OMRs. This leads to the further implication of waste in the larger healthcare system. This study also proposed a methodology to measure the significance of information relevant to multiple practice settings in a large-scale cohort, which will be beneficial for future studies in other departments.

## Conclusion and Future Work

Due to technical and systematic barriers, there exists an underutilization of information from OMRs. This study identified clinical concepts contained in OMRs that are beneficial to Cardiovascular Medicine at Mayo Clinic. This work represents an initial step towards automated information extraction from OMRs originating from diverse healthcare providers. Finally, this study showed that clinically relevant information was received in the early stages of patient care at Mayo Clinic and stored in initial consultant notes.

As follow-up studies, we will expand this analysis to cover additional clinical specialties at Mayo Clinic. We will also compare the pattern of the digitally faxed materials against materials transferred digitally via HIE systems to identify any similar patterns. We will compare the performance of existing summarization tools to the results using the methods presented in this study. Furthermore, we plan to expand our scope to multi-site institutions to assess the generalizability of this approach to extract critical information on clinical narratives. Automated summarization of OMRs is needed to minimize clinical workload and convey relevant information in a timely fashion. Identification of salient information is an important step in development of a system for automatic summarization of clinical notes.

## Footnotes

1
https://cloud.google.com/vision/docs/ocr

2
https://github.com/tesseract-ocr/tesseract

3
https://www.abbyy.com/en-us/finereader

4 http://www.transym.com/

5
http://www.nlm.nih.gov/research/umls/
